# Localized Drug Delivery Systems: An Update on Treatment Options for Head and Neck Squamous Cell Carcinomas

**DOI:** 10.3390/pharmaceutics15071844

**Published:** 2023-06-28

**Authors:** Arvind Hariharan, Simon D. Tran

**Affiliations:** McGill Craniofacial Tissue Engineering and Stem Cells Laboratory, Faculty of Dental Medicine and Oral Health Sciences, McGill University, 3640 University Street, Montreal, QC H3A 0C7, Canada; arvind.hariharan@mail.mcgill.ca

**Keywords:** localized drug delivery, transdermal, transmucosal, head and neck squamous cell carcinoma, penetration enhancers, nanoparticles

## Abstract

Head and neck squamous cell carcinoma (HNSCC) is one of the most common cancers in the world, with surgery, radiotherapy, chemotherapy, and immunotherapy being the primary treatment modalities. The treatment for HNSCC has evolved over time, due to which the prognosis has improved drastically. Despite the varied treatment options, major challenges persist. HNSCC chemotherapeutic and immunotherapeutic drugs are usually administered systemically, which could affect the patient’s quality of life due to the associated side effects. Moreover, the systemic administration of salivary stimulating agents for the treatment of radiation-induced xerostomia is associated with toxicities. Localized drug delivery systems (LDDS) are gaining importance, as they have the potential to provide non-invasive, patient-friendly alternatives to cancer therapy with reduced dose-limiting toxicities. LDDSs involve directly delivering a drug to the tissue or organ affected by the disease. Some of the common localized routes of administration include the transdermal and transmucosal drug delivery system (DDSs). This review will attempt to explore the different treatment options using LDDSs for the treatment of HNSCC and radiotherapy-induced damage and their potential to provide a better experience for patients, as well as the obstacles that need to be addressed to render them successful.

## 1. Introduction

**Figure 1:**
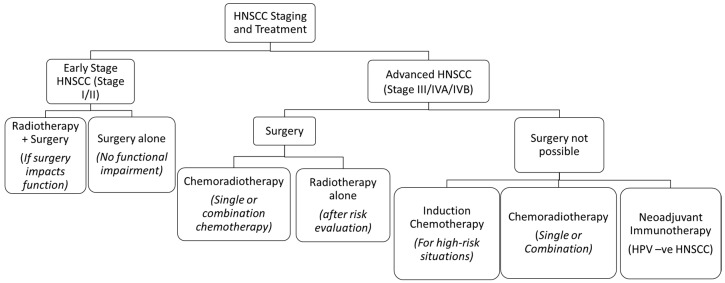
The treatment algorithm for HNSCC based on the stage of the tumour. *Reprinted and adapted from Seiwert* et al. [9,10].

Head and neck squamous cell carcinoma (HNSCC) is the sixth most common cancer in the world and has a multifactorial etiology [1]. Annually, HNSCC accounts for approximately 900,000 cases and 400,000 deaths globally [2]. Its treatment often warrants a multidisciplinary approach, which is dependent on the staging of the tumours [3]. Early stage HNSCC can often be managed by single treatment modalities like surgical resection or radiotherapy (RT), whereas a combination of chemotherapy, surgery, RT, and immunotherapy is used in locally advanced or metastatic stages [3,4] (Figure 1). Its treatment has evolved over time in all aspects, thus improving the prognosis and quality of life.

### 1.1. Chemotherapy

Chemotherapy (CT) is usually employed for locoregionally advanced HNSCC tumours, concurrently with radiotherapy either before surgery or as palliative therapy [5]. An important meta-analysis was conducted, to show that concurrent chemotherapy is the standard of care for locoregional HNSCC [6]. For these tumours, the most used drug is Cisplatin, which is either used alone or in combination with 5-Fluorouracil (5-FU), Carboplatin or Mitomycin-C [5,7,8].

Over the years, due to the toxicities associated with chemotherapeutic drugs, treatment regimens have undergone numerous modifications. An example of this is Induction Chemotherapy, where the main goal of treatment is to not disrupt the normal vasculature and to provide improved systemic tolerance, while eradicating tumour metastasis [11]. A trial was conducted in 437 patients with unresectable advanced HNSCC, which showed that the conventional three-cycle Cisplatin-5-FU regimen did not yield any survival benefits in comparison to 7 weeks of cisplatin and RT alone [12,13]. When Paclitaxel and Docetaxel were added, however, the results were superior, with reduced incidences of common side effects such as oral mucositis, neutropenia, and thrombocytopenia [13].

Despite improved treatment schedules, multiple controversies with systemic toxicities were recorded in subsequent trials [14]. The results from a series of randomized controlled trials did not show any additional benefits with induction chemotherapy, displaying weak response rates [14,15]. Also, patient sample sizes were inconsistent, therefore, adequate conclusions could not be formed [15]. These highlighted gaps in the literature necessitate newer methods of administering drugs to avoid systemic circulation.

### 1.2. Radiotherapy

Since its inception, radiation has played a key role in medicine. It remains the mainstay of cancer treatment, being prescribed 50% of the time [16]. Ionizing radiation acts on all living cells by transferring its energy to biomolecules either directly or indirectly through the by-products of water radiolysis on tissue to cause free radical formation and subsequent tissue changes and DNA damage [17]. Radiotherapy (RT) is usually given as internal (brachytherapy) or external beam RT [18]. The latter is provided through photons that are focused on the tumour site, whereas the former uses a radioactive seed placed close to the tumour [18]. Normally, RT is provided through fractionations up to a maximum total dosage of 70 Gy over a period of seven weeks [18].

Despite its evolution, RT still damages critical organs and tissues [17]. RT often damages tissues and organs by generating reactive oxygen species (ROS), which alters the DNA of the cells. Alteration in the cellular DNA causes mitotic catastrophe and leads to apoptosis [19]. The salivary glands (SG) are an example of critical organs that often lie in the path of radiation for HNSCC tumours [19]. Damage to the SG often results in dryness of the mouth (Xerostomia), which can often lead to debilitating conditions such as oral mucositis, dysphagia, and dental caries [19].

Although photons are commonly used in clinical settings, newer modalities are being used to treat HNSCC, which cause less damage to normal tissue. For instance, particle-based therapies, such as proton and neutron beam therapy, are known to reduce damage to tissues due to their unique physical properties [20]. Heavy particle therapy has undergone numerous randomized controlled trials for different cancers, including HNSCC. A prominent clinical trial investigated the performance of intensity-modulated proton therapy (IMPT) in comparison to conventional intensity-modulated radiotherapy (IMRT) and found that IMPT resulted in reduced severe weight loss and dependency on feeding tube in 50 patients after 32 months of follow-up [21]. Other heavy particles, such as in neutron beam or carbon ion beam therapy, have also been used on patients with HNSCC, especially in SG malignancies and soft tissue sarcomas [22,23]. Intensity-modulated neutron therapy (IMNT) has been shown to provide comparable tumour coverage for radioresistant HNSCC in comparison to IMRT, with superior sparing of healthy tissues and organs-at-risk (OARs) [24]. Heavy particles, however, need to be further investigated. There are studies that argue that although IMPT reduced side-effects to OARs, it is not clear whether there is any benefit for heavy particle therapy in comparison to IMRT when it comes to overall survival rates [25]. Also, heavy particle therapy is expensive and is only accessible to people with a high socio-economic status and in limited countries [26].

Other strategies to mitigate the harmful effects of radiation have been introduced over time, which have provided symptomatic relief but have not permanently resolved the damage caused. These strategies are also associated with their own side effects, which can affect patients undergoing RT for HNSCC. Considering the example of SG damage, the radioprotective drug Amifostine (AMI), for instance, was approved to relieve xerostomia in HNSCC patients due to RT [27,28]. It is a free radical scavenger, preventing the apoptosis of cells, and is usually given intravenously to patients prior to each cycle of fractionated RT [28,29]. It is often less preferred due to the associated systemic effects [29]. Other treatments to restore SG hypofunction are currently being tested, involving stem cell therapies like bone marrow cells and their extracts, for example [30]. But they are yet to materialize as bench-side treatment strategies and further research is required to avoid them being administered systemically. As is the case with chemotherapeutic agents, simpler, non-invasive, and localized methods of administration of agents such as AMI need to be considered in HNSCC settings.

### 1.3. Immunotherapy

HNSCC is associated with poor survival outcomes, especially where HPV-negative HNSCC is concerned. Advanced, recurrent, or metastatic disease is a great challenge to treat with chemoradiation and/or surgery alone. Immunotherapy is a novel addition to the treatment algorithm for most cancers including HNSCC. It works on the principle of activating an immune response in the host to eliminate tumour cells [31]. There are several types of immunotherapeutic agents that play a role in the immune pathways responsible for the interaction between cancer cell antigens and immune cells. The first immunotherapeutic drug to be evaluated was Cetuximab, which targeted the epidermal growth factor receptor (EGFR) pathway, a pathway vital for tumour cell survival and proliferation [32,33]. In 2006, Cetuximab secured FDA approval for advanced HNSCC based on trials showing its efficacy in combination with RT [34,35]. More recently, a new bi-weeklydosing regimen for Cetuximab was approved by the FDA for EGFR-expressing HNSCC, especially when used as a single treatment modality [36]. Studies have, however, shown that cetuximab may not be beneficial, especially in HPV-positive HNSCC [32]. Gillison et al. compared Cetuximab with Cisplatin in HPV-positive HNSCC patients and found that Cetuximab had inferior overall survival when compared to Cisplatin, after both sets of patients received 35 fractions of 70 Gy radiation [37]. Another trial by Mehanna et al. additionally demonstrated that Cetuximab did not reduce the overall toxicity in comparison to the standard Cisplatin regimen amongst the 334 patients that were treated, after 24 months of follow-up [38]. These ambiguities led to further research being conducted for newer immunotherapeutic agents.

The programmed cell death ligand-1 pathway (PDL-1) is the most exploited pathway by immunotherapeutic agents [10]. It is necessary to inhibit this pathway because it allows tumour cells to evade immune response when PDL-1 is upregulated [31]. In HNSCC, the first approved anti-PDL-1 agents were Nivolumab and Pembrolizumab [10,31,32]. A prominent clinical trial, known as Checkmate 141, was one of the first to evaluate the effects of Nivolumab on recurrent and/or metastatic HNSCC patients in comparison to the standard therapy (Methotrexate, Docetaxel, or Cetuximab) in 361 patients [39]. The investigators found that the overall survival and treatment response rate had improved with Nivolumab with fewer incidences of adverse effects [39]. A later, more recent trial compared the effects of Pembrolizumab with the same standard treatment regimen and found that the prolonged overall survival rate in HNSCC patients using pembrolizumab lead to it being used as a single therapeutic agent as well as in combination in early-stage disease [40]. These trials led to these agents being approved by the FDA for recurrent and/or metastatic HNSCC.

Over time, it was discovered that blocking the PDL-1 pathway became limited due to the reduction in the normal immune body responses, especially in the CD8^+^ T-lymphocytes [31,32]. The regulatory T-lymphocytes are attracted to the tumour micro-environment because of certain factors that suppress immune response [31]. Examples of these factors include interleukin-10 (IL-10), transforming growth factor-beta (TGF-ꞵ) which, in turn, express immune checkpoint proteins that can be further targeted [32]. The expression of immune suppression of these factors provides opportunities for testing immunotherapeutic agents on these new targets, which can further sensitize tumour response to anti-PDL agents.

Cytotoxic T-lymphocyte antigen-4 (CTLA-4) is a targetable immune checkpoint protein that is being exploited in clinical trials for HNSCC. An anti-CTLA-4 agent, Tremelimumab, was evaluated in a phase-III trial with 247 HNSCC patients, in comparison with the standard treatment regiment and Durvalumab, an anti-PDL1 agent [41]. The results found no statistically significant differences in either treatment groups in terms of overall survival; however, Durvalumab showed higher survival rates at 12 and 24 months and better clinical activity [41]. More clinical trials are currently recruiting HNSCC patients using Iplimumab, another anti-CTLA4 agent, and targeting other immune checkpoint proteins such as lymphocyte activation gene 3 (LAG-3) and mucin domain-containing protein 3 (TIM-3), all of which can potentially aid in the inhibition of the main PDL-1 pathway [42].

Innate immunity pathways are also being explored as potential targets. The stimulator of interferon genes or STING is an important pathway for regulating innate immune response. STING agonists were first tested in melanoma, colon cancer, and breast tumour mouse models to provide anti-tumour response [32]. In the HNSCC setting, a STING activator known as Cyclic Dinucleotide (CDN) has been tested in immunogenic mouse models to evaluate tumour growth inhibition [32]. Corrales et al. injected CDN intratumourally in immunogenic and non-immunogenic HNSCC mouse models and found that there was a 50% regression rate of the tumour in the former model [43]. Another recent study by Baird et al. incorporated a STING ligand into a hydrogel (Matrigel) and injected it intratumourally in HNSCC tumours resected from mice to demonstrate anti-tumour activity in comparison to controls [44]. These data suggest that the innate immune pathway could be targeted in further clinical trials.

### 1.4. Localized Drug Delivery Systems (LDDS)

**Figure 2:**
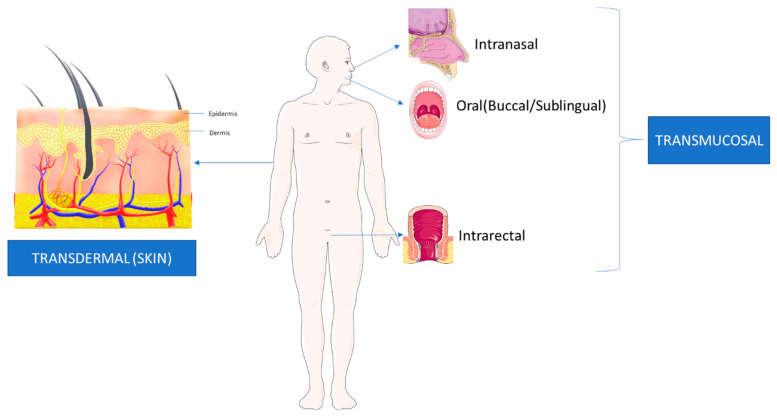
This figure shows the current methods of LDDS. Transdermal delivery involves agents that cross the skin layers before entering the area of interest. In transmucosal delivery, agents are delivered directly into mucous membranes, such as intranasal, oral, and intrarectal routes. *The Figure was partly generated using Servier Medical Art, provided by Servier, licensed under a Creative Commons Attribution 3.0 unported license* (www.smart.servier.com) (accessed on 5 April 2023).

LDDS are, “Formulations or devices that enable the introduction of a therapeutic substance into the body [45].” These systems are introduced locally through different routes of administration with a more rapid entry into the systemic circulation [45]. The rapid entry allows for a quicker onset of effects from these therapeutic agents, with fewer side effects. Research is also ongoing for more localized systems that avoid systemic circulation, thereby avoiding the associated toxicities of the drugs. LDDS revolve around the concept of, “Targeted Drug Delivery”, wherein a more direct access to the diseased organ or tissue is provided with minimal systemic circulation or metabolism of the drug [46,47]. LDDS are increasingly common for various cancers, including HNSCC, because they involve the addition of components to existing cancer therapeutics to adhere directly to the tumour cells on the endothelial surface [46]. Direct adherence is usually advantageous due to the lower interaction of the drug to non-targeted tissue [46]. However, the mode of administration also plays a role and further research is required to enhance LDDS by more localized drug delivery routes [45,47]. This manuscript will review the current applications of LDDS transmucosal and transdermal routes in HNSCC treatment as well as agents that reduce the effect of CT/RT-induced damage to tissues and organs. Figure 2 shows the difference between Transdermal and Transmucosal drug delivery.

## 2. Transdermal Drug Delivery

**Figure 3:**
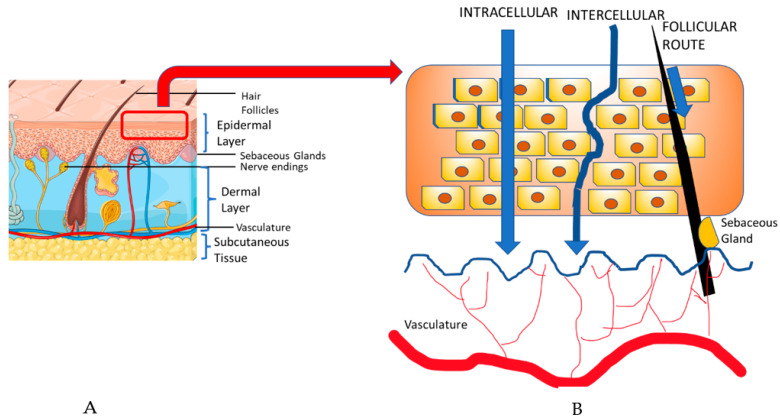
(**A**). The skin structure is composed of the epidermis, dermis, and subcutaneous tissue. (**B**). Transdermal drug delivery takes place in the stratum corneum layer of the epidermis and involves two pathways: the transepidermal and transappendegeal pathways. The transepidermal pathway involves molecules passing through the skin layers, either intracellularly or intercellularly, whereas the transappendegeal pathway (follicular route) involves delivery through other components present in the skin layer. *Adapted with permission from Alkilani* et al. [49]; *The Figure was partly generated using Servier Medical Art, provided by Servier, licensed under a Creative Commons Attribution 3.0 unported license* (www.smart.servier.com) (accessed on 5 April 2023).

Transdermal drug delivery (TDD) is a popular approach used to deliver drugs systemically through the skin [45]. In order to understand TDD, it is important to note the components of the skin. The skin consists of three layers, the epidermis, dermis, and the subcutaneous tissue. The epidermis consists of keratinocytes, dendritic cells, and other cell populations, all located in separate sub-layers, whereas the dermis is primarily composed of collagen, sebaceous glands, and vasculature [48]. TDD involves two possible pathways: the transepidermal and the transappendegeal pathways [49] (Figure 3). The transepidermal pathway involves molecules passing through the stratum corneum epidermal layer of the skin, either intracellularly (within cells) or intercellularly (between cells), for a prolonged period and the route can be used both systemically and locally. Transappendegeal pathways (follicular route) involve drugs that are delivered through sweat ducts, sebaceous glands, and hair follicles before entering systemic circulation. Although it is a systemic route, it is often preferred because of the less invasive and more localized nature compared to the conventional parenteral route [45,50]. Usually, the transdermal approach uses agents in the form of transdermal gels or patches containing different penetration enhancers (PE) or drug carriers [45]. The introduction of PEs and drug carriers are particularly significant in cancer therapies, due to the impenetrable nature of the anti-cancer macromolecules [51,52].

### 2.1. Transdermal Gels

The term, ‘gel’ was coined in the 1800s to distinguish materials that are usually semi-solid in nature based on their pharmacological classification [45,53]. According to the United States Pharmacopeia (USP), gels are semisolid systems consisting of the dispersant inorganic substances that are enclosed with liquid [53]. They are three-dimensional matrices that are made up of polymers dispersed throughout [53]. They are popular due to their swift cutaneous absorption and ease of application and are used in pain management, testosterone replacement therapy, and chemoprevention [54,55,56].

Transdermal gels have a wide range of applications related to cancers, ranging from pain management to direct chemoprevention. Chemotherapy induces debilitating effects, such as nausea and vomiting, and, despite the usage of antiemetic drugs, it continues to be a significant barrier to the treatment [56,57]. Bleicher et al. conducted a series of trials investigating a topical gel containing Lorazepam, Diphenhydramine, and Haloperidol to rescue a total of 33 cancer patients undergoing chemotherapy from nausea and vomiting [56]. These trials suggested that the topical gel was able to suppress nausea without any associated side effects, thus appearing to be a promising therapeutic intervention.

Transdermal gels have shown a wider application in HNSCC pain management, as well as in RT-induced oral mucositis and dermatitis. During HNSCC chemoradiotherapy, incidences of pain due to radiation-induced dermatitis can be debilitating for the patient [58]. Early studies have shown that opioid receptors play a role in the modulation of pain, thus leading to numerous studies using morphine gel on ulcers [59]. A series of case reports in Japan were the first to record the use of topical morphine for HNSCC patients suffering from dermatitis and tumour infiltration [60]. They showed that pain control was maintained in all the cases, while controlling the adverse effects of the initial pain management using systemic administration [60]. A later randomized controlled trial evaluated topical morphine for cancer-related painful mucosal and cutaneous lesions and found it to be fast-acting, effective, and safe for reducing pain intensity in comparison to the placebo treatment [61].

Oral mucositis (OM) is the inflammation of the oral mucosa, usually associated with a burning or tingling sensation and is one of the main adverse effects of chemoradiotherapy for HNSCC [62]. Although there is extensive literature investigating the management of OM, there is only one drug, Palifermin, that is currently approved by the FDA [63]. However, there is still no medication that can prevent radiation-induced OM and that is a cause for concern. Research has shown potential candidates that can be given topically to reduce adverse systemic effects [64]. Tetracaine gel was tested in a phase II trial to assess pain relief for radiation-induced mucositis, which showed a 79% reduction in pain and no incidences of side effects for 82% of patients [65]. Later studies followed up on investigating the option of using transdermal gels for OM. For instance, a French group of investigators developed a topical thermogel containing active metabolites of Amifostine and found that, when applied around the lip region in mice, the severity of OM was adequately reduced [66]. Newer agents tested included a cream containing turmeric and sandalwood oil for radiation-induced dermatitis, with promising results for prevention [67]. While there is potential for further research in this area, plans should be made to validate these agents in larger trials.

Amongst the critical organs and tissues that RT affects during HNSCC treatment, the SGs are commonly affected. SG hypofunction causes xerostomia which, in turn, leads to oral conditions such as dental caries, dysphagia, and loss of bone [19]. This reduces the patients’ quality of life, thus necessitating therapeutic interventions to alleviate SG hypofunction. Transdermal gels have not been commonly explored for SG hypofunction; however, Cotrim et al. investigated the radioprotective drug, Tempol, as a topical gel for the prevention of Xerostomia and found that it was as effective in radioprotection when compared to other conventional routes [68,69]. However, there are no further studies to verify these findings.

Caries management is also essential during RT, as SG hypofunction can lead to the demineralization of the tooth structure [70]. The use of topical fluoride has become the mainstay of practice for HNSCC patients. The first recorded use of fluorides was by Dreizen et al. in 1977 using custom trays [71]. However, patient compliance was an issue, especially due to the lack of patients returning to the clinic [72]. Also, although it is an approved standard of care, there is still ambiguity surrounding the optimum fluoride concentration required for application, necessitating more randomized controlled trials [70].

Salivary substitutes have been shown to be effective in alleviating xerostomia; however, because they cannot be consumed orally, their use in dysphagia is currently under investigation [73]. A novel edible oral mucosal jelly was synthesized in Thailand and studies showed that it had potential to reduce xerostomia in patients with hypertension [73]. Nuchit et al. investigated its potential for aiding swallowing ability in 66 patients post-irradiation for HNSCC and compared it with a commercially available topical saliva gel [73]. They found that, if given on a regular basis, topical salivary substitutes could improve xerostomia in HNSCC, while alleviating dysphagia [73]. While salivary substitutes do not provide permanent relief from SG hypofunction, their topical application could be used as first-line therapy to provide acute relief from xerostomia without systemic side effects.

### 2.2. Transdermal Patches

TDD systems commonly come in the form of transdermal patches that can be modified with PEs. It is an adhesive patch that contains medicaments placed over the skin to deliver specific dosages into the bloodstream [74,75]. The basic components of a typical transdermal patch include: (a) the Polymer matrix, which controls the drug release, (b) the drug itself, (c) penetration enhancers (PEs) to increase the skin permeability, and (d) other components such as adhesives, backing laminates, release liners, plasticizers, and solvents [76]. The first transdermal patches were approved by the FDA in the US in 1979 for Scopolamine to treat nausea and vomiting [74,75]. This was followed by the approval of nicotine patches in 1984 and then further patches for pain management and hormone replacement therapy [74]. The technology of transdermal patches continues to evolve for a wider range of medical problems, including head and neck cancers.

Transdermal patches have been investigated in pain management in different cancers using fentanyl [77]. A systematic review by Hadley et al. examined various reviews analyzing the effectiveness of fentanyl transdermal patches on cancer pain and, although they found that they significantly relieve cancer pain, they acknowledged that the literature review was limited and most of the studies that were considered had very few participants [78]. A later systematic review compared transdermal patches of fentanyl and another opioid, buprenorphine, and found them to have similar efficacy, although they concluded that the latter could have reduced risk of tolerance [77]. This increases the number of options for transdermal patches for cancer pain management.

There is evidence that transdermal patches are preferred in patients with head and neck cancer to control baseline pain, since oral medication could be difficult to administer [79]. An early study examining the time trends and characteristics of prescribing opioids for patients with a variety of cancers, showed that fentanyl was prescribed 10.2% of the time during the last 3 months, particularly in head and neck cancer cases using transdermal patches on most occasions. The efficacy, safety, and quality of life of head and neck cancer patients using fentanyl transdermal patches were also evaluated in a variety of prospective studies to show a significant decrease in pain, a better quality of life, and reduced side effects, the most common being nausea and vomiting [80,81,82]. However, there is a lack of studies that compare Fentanyl transdermal patches with other opioid analgesics [79]. One notable randomized controlled trial by Haumann et al. was performed on 52 head and neck cancer patients experiencing severe neuropathic pain [83]. Fentanyl transdermal patches were compared with oral methadone in 52 patents and it was found that the latter performed better in pain management, but there were no significant differences in the alleviation of side effects [83]. More such studies are required to assess the performance of fentanyl patches in comparison with standard opioids.

### 2.3. Penetration Enhancers

**Figure 4:**
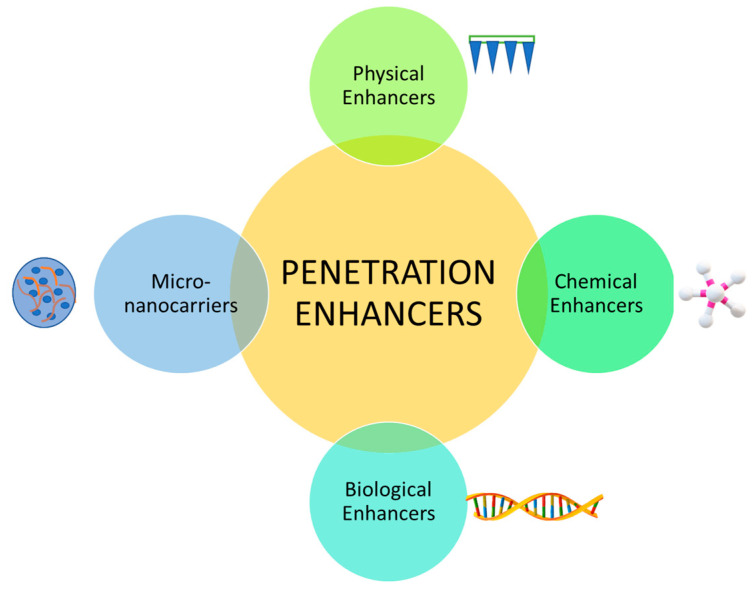
The difference penetration enhancers that have been explored are: 1. physical (e.g., microneedles, iontophoresis, electroporation), 2. chemical (e.g., alcohols, dimethyl sulfoxide (DMSO), alkanols, and surfactant), 3. biological (e.g., peptide-based), and 4. micro-nanocarriers (e.g., inorganic nanoparticles, liposomes) [51].

Penetration enhancers (PEs) are necessary for modern TDDs because the skin has a heavily protective barrier that prevents unwanted substances entering the body [52,84]. Although these barriers are effective, they do not allow many TDD to provide any therapeutic effects. PEs do not have any therapeutic properties of their own; however, they play a significant role in the transport of existing drugs through the skin without any toxicities [84]. For transdermal patches involved in cancers, there are different PEs that have been explored (Figure 4) [51].

#### 2.3.1. Chemical and Biological Enhancers

Chemical enhancers improve drug penetration by regulating the obstacles that affect penetration of the stratum corneum [51]. Most mechanisms involve the altering of the lipid structure, interacting with the intracellular proteins as well as altering the fluidity of the stratum corneum bilayer [51,84]. Research has shown the potential of chemical enhancers in altering skin structure. For instance, in vitro studies on various cancer cell lines have evaluated the use of essential oils as chemical permeation enhancers to provide anti-tumour activity and they have been found to inhibit tumour growth [85,86]. However, skin irritancy is a common side effect due to the alteration of skin structure, especially since copious amounts are required to produce better permeability [51]. The increased potential of skin irritation by chemical enhancers has contributed to limited studies in cancer settings [51]. Newer technologies are being investigated, involving the incorporation of other PEs such as micro-nanocarriers and physical enhancement techniques, to provide a safer experience for patients.

Most biological enhancers are peptide-based and are effective carriers of substances such as proteins, polymers, nanoparticles, and nucleic acids [51]. These peptides have been shown to enhance TDD by delivering drugs such as insulin, growth hormones, and sodium diclofenac for easier absorption into the bloodstream [51]. The mechanism of action of these biological enhancers remains unclear; however, a possible suggested mechanism is that they work by opening the skin channel to allow the proteins to reach the blood vessels [51,87]. They have also been used in melanomas in combination with micro-nanocarriers like liposomes and transferosomes to successfully deliver chemotherapeutic drugs like paclitaxel and vemurafenib [88,89]. However, further research is required to understand the mechanism of action of biological enhancers for regular use in HNSCC settings.

#### 2.3.2. Micro-Nanocarriers

Micro-nanocarrier systems have been used increasingly for TDD, including transdermal patches. They enhance the permeability by increasing the solubility of drugs [51]. Micro-nanocarriers are being explored in cancers to deliver chemotherapeutic agents to improve solubility and controlled drug release [51]. They have been used to provide treatment to localized skin cancers, with improved target efficiency and reduced systemic toxicity; however, their use in HNSCC is still yet to be explored. Table 1 shows the various micro-nanocarriers that are used in different cancer settings.

Vesicles are drug delivery vehicles that have been used in TDD systems. They are colloidal particles that contain water in a bilayer arrangement and have the capacity to carry both water-soluble and fat-soluble drugs [95]. Vesicles are either engineered synthetically (e.g., liposomes) or are isolated naturally from cells (extracellular vesicles) [52,103]. Examples of vesicles that have been used extracellularly are exosomes, which are gaining importance as potential drug delivery vehicles in cancers in transmucosal drug delivery [104].

Liposomes are soft vesicles composed of phospholipids, which are formed by bilayer membranes that can separate between multiple aqueous medias [95]. They can stay only on the surface of the skin, which can be advantageous because it minimizes the amount of drug that enters the bloodstream and allows for sustained release long-term [95]. Owing to this feature, it is often preferred for topical application, especially for skin diseases. Liposomes were first approved in the form of Doxorubicin (Doxil) in 1995 for ovarian cancer, kaposi’s sarcoma, and multiple myeloma when given intravenously [90]. They have also found application in HNSCC with reasonable success. Harrington et al. conducted a Phase-II trial for 20 HNSCC patients, where two cycles of pegylated liposomal-Doxorubicin was given every 3 weeks prior to RT [91]. They found low incidences of hematological, mucosal, and cardiac toxicities [91]. Another in vivo study using liposomal-Doxorubicin on HNSCC xenografts in mice showed a prolonged circulation time of the drug so that it could be accumulated into the tumour [92]. The inference from this result is that liposomes allow for good bioavailability and tumour localization. However, the application of liposomes is still limited due to the decreased encapsulation efficacy and short shelf life [95]. Modifications of liposomes have been tried on various occasions to deliver chemotherapeutic agents, such as transferosomes and ethosomes, that allow for greater stability and better retention of the drug [95]. Transferosomes, in combination with biological enhancers, have been investigated with Doxorubicin for lymphatic delivery of the drug to avoid liver and kidney metabolism [105]; however, the focus was shifted to ethosomes due to their superior stability [95].

Other nanoparticles (NPs) that have been used for cancer therapies include inorganic NPs and polymeric NPs [95,106]. Polymeric NPs allow the drug to be attached to the exterior of the NP to regulate the release of the drug [95,107]. Initially, they were made of non-biodegradable polymers; however, due to their toxicity to the body systems, biodegradable polymers such as Chitosan, Albumin, and Alginate were introduced. The albumin-based NP, Abraxane, has especially been gaining traction for offering anticancer treatment with lower cardiotoxicity [90]. Further additions of chemical enhancers have also proved to improve interactions with endothelial cells located in the blood–brain barrier [51]. Polymers have been tested with drugs such as Paclitaxel, Doxorubicin, and 5-FU for various anticancer therapies [96]. Polymeric NPs containing Cisplatin and Paclitaxel are undergoing trials and concrete conclusions are yet to be drawn and they are yet to gain approval for HNSCC [98,99]. Also, most clinical trials that are underway using polymeric NPs involve intravenous and intratumoural administration and more research is required for their use in TDD systems for HNSCC [96]. The intratumoural administration of NPs is discussed later in this review.

Inorganic solid NPs, such as Gold NPs, Carbon NPs, and other metallic and magnetic NPs, have also widely been investigated as drug delivery vehicles for skin cancers [107]. The metallic and magnetic NPs are mainly used in imaging for diagnostic purposes as well as in combination with physical enhancement techniques such as thermal ablation [107]. For HNSCC treatment, the objectives for the use of NPs lie in enhancing the effects of radiotherapy as well as performing targeted drug delivery [96]. Numerous clinical trials and in vitro tests are underway to investigate the safety and efficacy of inorganic NPs for HNSCC, especially OSCC; however, based on the limited data, it is likely to be a long time before they are introduced for TDD systems [96].

#### 2.3.3. Physical Enhancers

Physical enhancers have been gaining popularity due to their ability to temporarily modify the skin integrity for drug release [51]. They are known to have greater efficiency than the regular topical delivery of drugs due to their more rapid delivery [95]. Usually, physical enhancers can either be energy-driven or electrically driven devices for enhancing drug absorption through the skin [108]. Some common physical enhancement techniques that are used are iontophoresis, sonophoresis, electroporation, thermal ablation, and microneedles.

Iontophoresis involves the movement of ions across the cell membrane through the application of a mild potential difference [95]. Drug permeation is increased into the skin through the usage of mild electrical currents [95,108]. This enhancement technique has been employed for the delivery of anti-inflammatory drugs, for cosmetic skincare, and with limited applications in cancer therapy [108,109]. Iontophoresis has been widely used for skin cancers especially. An in vivo study by Petrilli et al. showed that topical iontophoresis increased skin cancer treatment effectiveness through the usage of EGFR-targeted liposomes containing 5-FU [110]. They found that it was more effective in comparison to subcutaneous injections of 5-FU with liposomes [110]. Iontophoresis has also been tested for HNSCC on in vitro bovine mucosa, where chemotherapeutic drugs like 5-FU and Leucovorin were delivered using buccal iontophoresis, which increased the deposition of both drugs [111]. An ex vivo study using chitosan NPs showed that the addition of iontophoresis doubled the amount of drug released from the NPs on oral tumour cell lines [112]. These studies highlight the potential of iontophoresis in HNSCC and therefore warrant further clinical investigation.

Sonophoresis is another method of TDD that involves the transport of drugs through the skin using ultrasound [95]. It acts by causing gaseous cavitations within cells while simultaneously elevating the skin temperature for drug release [95]. This method has been tested with various drugs such as insulin, ketoprofen, vancomycin, and various proteins in vivo; however due to limited results and a limited understanding of the mechanism of action, it is yet to be tested in humans [108]. Sonophoresis has also been investigated for skin and cervical cancer xenograft models with chemotherapeutic drugs such as cisplatin [113]. It has not been directly used for HNSCC; however, low frequency sonophoresis with fibroblast growth factor has been proposed as an adjunct to surgery in the treatment of osteoradionecrosis of the jaw [114].

Electroporation is another physical enhancement technique, which is the process of inducing pores in cell membranes by the application of electric currents through short electric pulses [95]. It has been shown as a potentially effective TDD system for delivering drugs such as insulin and different vaccines, but has also been tested with chemotherapeutic drugs in cancers [108]. In HNSCC, a phase I study involving six patients with recurrent HNSCC used calcium electroporation [115]. Calcium was administered intratumourally, followed by electroporation, and was found to be safe and feasible, thus warranting further studies [115]. Other studies have used intratumoral injections of Bleomycin with electroporation and found it to be a valid treatment option [116]. Further studies are required for evaluating the usefulness of electroporation as a TDD system, because it is associated with limitations such as high equipment costs, complicated use, and being time consuming [108]. Research has also shown that electroporation can cause cell death and drug damage due to denaturation [108].

A simpler enhancement technique is Thermal Ablation, which involves the application of heat through transient electric pulses locally to create microchannels on the skin surface for drug release [95]. It is usually induced by laser and radiofrequency methods to enhance the delivery of both lipophilic and hydrophilic drugs and vaccines [95]. There are instances where thermal ablation has been used in HNSCC treatment alongside radiation devices like MRI-guided lasers and microwave ablation [117,118]. Studies have shown that it can be performed with high accuracy and safety when compared to surgery because it does not require any incisions and there is minimal blood loss. The procedure is also rapid, taking less than an hour on average [95]. A study by Melancon et al. used MRI-guided laser ablation to deliver monoclonal antibodies coated with gold nanoparticles and found that they were effectively delivered to HNSCC tumours both in vivo and in vitro [118]. A later study evaluated the safety and efficacy of ultrasound-guided microwave ablation for the palliative treatment of HNSCC malignancies, in a sample size of 18 patients, and found that it not only provided safe and effective treatment, but also accurate monitoring of the ablation site and surrounding tissues [117]. This technique can therefore be tested with TDD systems as well to evaluate the intratumoural entry of drugs.

**Figure 5:**
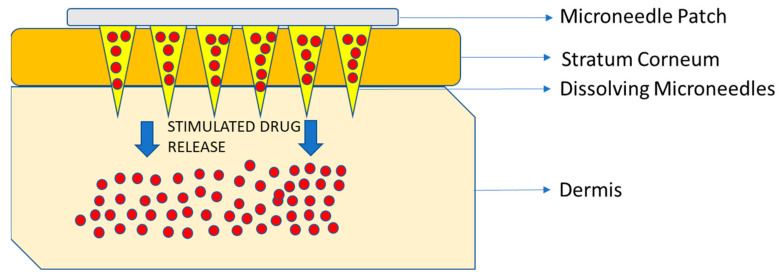
Schematic diagram of the microneedle patch. The base of the patch is normally made of a polymeric matrix that has dissolving microneedles containing the agent, attached to it. Once the needles pierce the stratum corneum, the needles dissolve due to internal or external stimuli, that causes drug release through the dermis to the area of interest and systemic circulation. *Information obtained from* [51,95,119].

Microneedles (MNs) embedded in transdermal patches have been tested and used in various settings successfully [95]. MN drug devices were first patented in 1976 and then, in 1997, the first MN for transdermal delivery was proposed [108]. They are the most widely investigated physical enhancers for drug and vaccine delivery because they can easily penetrate the stratum corneum painlessly without irritating the nerve endings and allowing for rapid absorption into the surrounding capillaries and lymph nodes [108]. The most common types of MNs that are being used are dissolving and hydrogel-forming MNs for drug delivery [108] (Figure 5).

MNs have been investigated for anticancer therapies, most commonly for skin melanomas and subcutaneous tumours [51]. There are studies of drug delivery using MNs in immunotherapy, gene therapy, phototherapy, and a combination of therapies [51]. Their usage in HNSCC remains scarce; however, a notable study by Lan et al. showed that MNs could be an effective way of delivering anticancer drugs [120]. MNs containing nanoparticle-loaded cisplatin were tested in vitro on HNSCC cancer cell lines as well as in vivo on HNSCC tumour mouse models [120]. The study found that MNs were safe and efficient in regulating the drug release, as well as in allowing cisplatin to provide its cytotoxic effects with reduced systemic toxicity [120]. The same group of researchers also found that, through combining cisplatin with immunotherapy using the same MNs, they were able to enhance the immune response against HNSCC in vivo and in vitro [121]. Another study used dissolving MNs with anti-CTLA, an antibody frequently used in HNSCC treatment [122]. In transgenic mice, they demonstrated that MNs could prevent the drug from being dispersed systemically, as well as protect them from immune-related adverse events [122]. They also found that MNs were just as effective in providing antitumour response in comparison to the systemic administration [122]. These studies show the potential of MNs and their uses in HNSCC and warrant further studies, especially since there is evidence that larger MNs can be created for use in patients [123].

## 3. Transmucosal Drug Delivery

Mucous membranes are those structures that cover all the internal passages and orifices in the body [45]. Due to this feature, drugs can be delivered locally at various sites across mucous membranes [45,124]. The mechanism of action by which mucous membranes facilitate drug delivery is known to be due to diffusion [124]. Diffusion allows for a constant, fixed amount of substance to cross the membrane at a particular time; however, it does come with its challenges [125]. Many drugs containing peptides or proteins cannot cross the mucosal barriers and are often degraded by the time they reach the bloodstream, thereby necessitating the addition of PEs such as bile salts, fatty acids, or phospholipids [124,125]. Transmucosal routes utilize two different pathways for drug transport. Once drugs reach the mucosal surface, they cross the epithelium either through the cells (transcellular route) or between adjacent cells (paracellular route), depending on their physicochemical properties [118]. Table 2 shows the types of routes and their applications. Each of the routes have unique properties but the common factor amongst them is the presence of a mucous layer that can facilitate drug delivery [45,124].

### 3.1. Intranasal Delivery

The intranasal route is widely used for the management of localized symptoms such as nasal congestion and rhinitis [124]. The nasal cavity has a large surface area, which allows for an extensive vascularization network [124]. Due to this extensive network, the intranasal route is becoming a more sought-after mode of administering drugs systemically for various uses, ranging from cancer pain management to bleeding control, and are dispensed mainly as sprays or powders [125,127]. A study by Mercadante et al. compared the intranasal fentanyl spray with the oral transmucosal fentanyl citrate in 196 adult cancer patients and found that there were significantly more patients that attained pain relief with the intranasal spray [127]. A subsequent investigation by the same authors confirmed that the long-term use of intranasal fentanyl in advanced cancer pain management was safe and effective without any adverse effects [126]. Various modifications in the formulations of fentanyl sprays were studied to prolong nasal mucosal contact for providing an extended analgesia effect. Studies showed that the addition of pectin in the nasal sprays were able to demonstrate reduced drug levels in the plasma in comparison to non-pectin containing nasal sprays [134,135]. The pectin intranasal delivery method has been tested in the head and neck cancer setting. Mazzola et al. conducted a trial on 40 head and neck cancer patients with painful mucositis using fentanyl pectin nasal spray and found that it was adequate in reducing mucositis with fewer adverse effects [136]. There are no trials in the literature, however, with a bigger sample size or any comparison treatment group. Chitosan NPs were also used to encapsulate fentanyl for intranasal delivery effectively to allow the drug to easily penetrate the nasal mucosa, which could potentially be useful in HNSCC [137]. Intranasal drug delivery, however, does come with limitations, such as limited drug dissolution and dosages, the presence of enzymatic activity in the nasal mucosa, as well as local irritation [124].

### 3.2. Rectal Delivery

Rectal delivery is mainly used for very localized conditions, with some instances of usage for colon cancer [124,132]. The highly vascularized environment allows for rapid systemic absorption in case of emergencies [124]. It is also a preferred route for patients because there are no chances of vomiting through this route, as opposed to the oral route [133]. The rate of absorption of the drugs depends on the area of absorption, for example, whether it is the lower or upper rectum [124]. The uses for rectal delivery lie in pain management, sedation, and treatment of seizures. It is not commonly used in palliative care, except for pediatric practice [124].

Novel rectal drug delivery systems have been investigated for the treatment of colon cancers. Chitosan-based drug carriers have been tested as rectal drug delivery vessels to treat conditions such as ulcerative colitis which, when treated, could prevent malignant transformation [138]. Chemotherapeutic drugs such as 5-FU and Docetaxel have also been encapsulated in drug carriers to be delivered rectally. Seo et al. used docetaxel-loaded nanomicelles for anti-tumour efficacy and found that they demonstrated the ability to improve the bioavailability of the drug and increased the chemotherapeutic potential [139]. A more recent study used a gelatin-based hydrogel containing 5-FU for rectal administration in colon adenocarcinoma cell lines successfully; however, it was an in vitro study and warrants further investigation [140]. Other drug carriers, such as carbon nanoparticles and liquid suppositories, have been investigated for delivering anti-cancer drugs, with improved outcomes [141,142]. However, the rectal delivery method in general has gone out of favour due to the associated limitations, such as concerns about the lack of privacy and acceptance by patients and clinicians, drug dissolution in the rectum, and leakage [124].

### 3.3. Oral Delivery

The oral transmucosal route involves the local and systemic delivery of drugs through the mucous membrane in the oral cavity and is a very popular route due to the ease of administration and convenience. This method of administration has been available since the early 1980s and usually involves drug delivery through buccal and sublingual routes to enter local or systemic circulation [143]. The buccal route is often preferred over the sublingual route because of reduced permeability to prevent leakage of drugs systemically [143,144]. To understand the oral route, it is important to understand the structure of the oral mucosa. The oral mucosa consists of stratified squamous epithelium, with connective tissue components, that is separated by a basement membrane [46,145]. The basement membrane is replenished by basal keratinocytes, which lie adjacent to each other [145]. This structure of the oral mucosa poses a challenge for transmucosal drug delivery due to the thickness of the mucosal layer [144,145]. It is especially challenging in patients who undergo cancer treatment because they are associated with conditions such as xerostomia and oral mucositis [144]. Also, specialized conditions are required to facilitate the permeability of the drugs such as in terms of the pH, fluid volume, and permeability of the mucosa [144]. The primary mechanism of action of drug delivery is through passive diffusion across lipid membranes, either paracellularly or transcellularly.

Drug technologies have improved to facilitate drug permeation through the oral mucosa. Initially, tablets and lozenges were the main forms of drugs that were used; however, this evolved further to sprays, patches, hydrogels, microspheres, and other drug delivery vectors [146]. Currently, these technologies have further improved with the introduction of PEs, mucoadhesives, and nanoparticles. Also, lowering the enzymatic activity facilitates drug delivery. There are numerous studies that have investigated the use of these technologies in HNSCC settings.

### 3.4. Oral Delivery—HNSCC

Oral transmucosal DDS are commercially available in many different forms; however, in the case of HNSCC, the options are more limited. In general, applications for HNSCC are mainly for pain management, followed by radiation-induced xerostomia and oral mucositis. More recently, the oral route has also been investigated for anti-tumour response in HNSCC. Table 3 enumerates the various applications and methods of oral transmucosal drug delivery in HNSCC.

#### 3.4.1. Pain Management

Just like other cancers, HNSCC is often associated with severe breakthrough pain, experienced in up to 80% of patients [79]. Opioid treatment is generally preferred for pain management in cancer patients; however, the ability for these patients to ingest certain drug formulations are limited [164]. Fentanyl is a very popular opioid because of the rapid onset and short duration of action [165]. It is also preferred because it is known to be more potent than other opioids [79]. It is available in different formulations as well, each of which provides effects against cancer breakthrough pain.

One of the premier clinical trials in 2008 tested the safety of a mucoadhesive form of Fentanyl [166]. A randomized controlled study and an open-label study were conducted simultaneously on patients who had cancer breakthrough pain [166]. These studies found that Fentanyl was safe and well-tolerated amongst the patients and was a good option to use as an opioid-based analgesic [166]. There is still a dearth in the literature, however, regarding the use of oral mucoadhesive Fentanyl in HNSCC due to the preference for the intranasal routes of delivery as well as poor representation of the population [79].

#### 3.4.2. Radiation-Induced Xerostomia

For the management of xerostomia, many of the agents that have been tested for saliva production used the transmucosal approach. For instance, Pilocarpine, the most commonly used agent, is available in transmucosal formulations, such as buccal inserts, hydrogels, and hyaluronate sheets [167]. Pilocarpine lozenges and mouth sprays have also been investigated and have shown potential in alleviating xerostomia and in safety [167]. The earliest study to use a topical form of Pilocarpine for radiation-induced xerostomia was a clinical trial on 20 patients conducted by Davies et al., wherein they compared a Pilocarpine mouthwash with artificial saliva [147]. They found that the mouthwash was more effective than the artificial saliva [147]. This led to further clinical trials using Pilocarpine mouthwash and evaluating its safety and efficacy.

There are studies, however, that have argued that these methods of administering Pilocarpine seemed to mainly target the minor SGs and that alternative methods targeting the major SGs are required [167]. Attention has turned towards using mucoadhesive polymers and hydrogels incorporating Pilocarpine. One of the first studies by Gibson et al. incorporated Pilocarpine in a hydrogel buccal insert for 8 patients with Sjögren’s Syndrome, and found that salivary and lacrimal secretions had increased, and the inserts were well-tolerated by patients [148]. These results led to studies using mucoadhesive polymers in radiation-induced xerostomia. More recently, chitosan was introduced as a mucoadhesive due to its superior adhesive properties for the buccal mucosa [149,168]. Another pivotal study by Muthumariappan et al. used Pilocarpine-loaded polymer nanofiber mats for local delivery to the SGs and found that these mats allowed for a controlled release of the drug as well as increased salivary secretion in vivo [150]. All these studies have showed the potential of using these new transmucosal methods for Pilocarpine and further studies are required to evaluate their use clinically.

Using transmucosal methods, other salivary stimulants and radioprotective drugs have also been studied. Clinical trials with mucoadhesive dietary supplements, were initiated for the treatment of radiation-induced xerostomia but had to be terminated due to low recruitment [151]. However, recent studies did show that the dietary supplement, Aqualief, was able to restore salivary secretion with reduced adverse effects; however, further studies are required due to the unsuccessful accrual of subjects [152]. Newer therapeutic methods for delivering Xylitol, another common salivary stimulant, are also being considered. For example, a recent study by Elkanayati et al. investigated buccal mucoadhesive films containing Xylitol and Adipic Acid, that were manufactured using 3D printing and hot melting techniques [153]. They found that these films demonstrated a uniform release of the agents in vitro, which shows that they can be used as alternative methods to deliver salivary stimulants [153].

#### 3.4.3. Oral Mucositis (OM)

OM treatment options are generally very sparse, apart from approved drugs to provide symptomatic relief. Various clinical trials are underway to use these drugs in forms which involve transmucosal administration. The mucoadhesive hydrogel, known as MuGard, was investigated in various clinical trials to mitigate OM symptoms, and was found to be superior in alleviating symptoms of OM and delaying the progression compared to regular oral rinses [154,155,156].

Another clinical trial compared Triamcinolone Acetonide, a common corticosteroid used to relieve mouth ulcers present in OM and Licorice, a natural product known for their effectiveness against oral diseases in 60 patients with OM [157]. The idea of this trial was to not only compare two agents but to evaluate their performance when integrated into mucoadhesive films. The investigators found that mucoadhesive films containing both drugs were equally effective to manage OM caused due to radiotherapy, with licorice providing a slightly better reduction in oral discomfort [157].

Mucoadhesive buccal tablets have also been used for the treatment of OM. Clonidine Lauriad is a drug that had been fast-tracked for investigation by the FDA in 2014 for the treatment and prevention of OM in cancer patients [158]. Originally known for its anti-hypertensive activity, Clonidine was first tested on hamster models for OM and found that it reduced its severity and duration [158]. Due to this, clinical trials using mucoadhesive buccal tablets were conducted. Giralt et al. conducted a series of trials in 2015, and more recently in 2020 with Clonidine mucoadhesive buccal tablets, and found that they could reduce the severity and duration of OM; however, further evaluation is required as the primary endpoints were not completely achieved in the trials [159].

Mucoadhesive topical gels have also been used for alleviating the symptoms of OM. Chamomile is a natural herb that is known for its anti-inflammatory properties and was frequently investigated with mucoadhesive topical gels [25]. One of the pivotal studies by Braga et al. showed that mouthwash containing Chamomile extract was able to reduce the severity, incidence, and duration of OM, caused due to allogenic hematopoietic stem cell transplantation in 40 patients [25]. This study inspired further studies using modified transmucosal techniques using Chamomile. A clinical trial by Elhadad et al. on 45 patients undergoing chemotherapy showed that a topical gel of Chamomile reduced the severity of chemotherapy-induced OM in comparison to conventional anti-fungal agents, topical anaesthetics, and anti-inflammatory sprays [160]. These transmucosal methods of administering drugs that can alleviate symptoms of OM have shown great potential and warrant further investigation.

#### 3.4.4. Anti-Tumour Response

**Figure 6:**
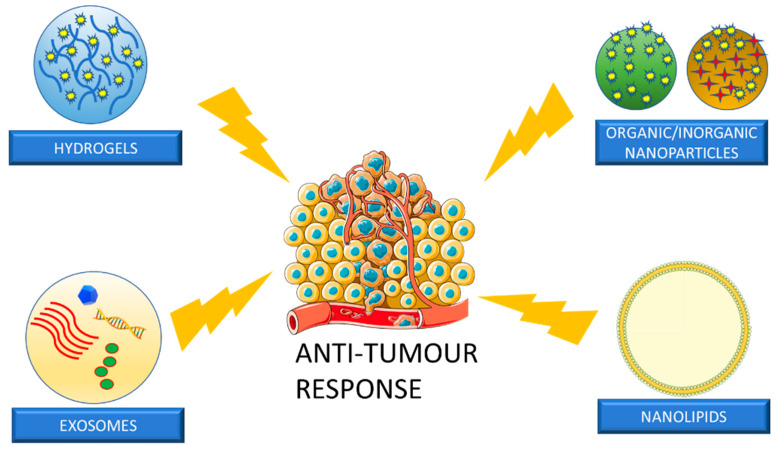
For HNSCC anti-tumour response, common nanoparticles that have been used are hydrogels, nanoparticles, nanolipids, and exosomes. *The Figure was partly generated using Servier Medical Art, provided by Servier, licensed under a Creative Commons Attribution 3.0 unported license* (www.smart.servier.com) (accessed on 5 April 2023).

Although chemoradiation, immunotherapy, and surgical techniques have evolved over time in the treatment of HNSCC, they still lead to serious adverse effects, which affect the patient’s well-being [161,162]. Therefore, it is essential to investigate novel therapeutic approaches to target the tumour simultaneously with early detection. Transmucosal DDS are starting to play a significant role in anti-tumour drug delivery. Conventional agents can be applied locally onto the tumour, as well as delivered through carrier-based approaches. Carrier-based approaches are advantageous because they possess the ability to control drug release as well as improve safety and efficacy [161,163]. Common carriers used in transmucosal DDS for HNSCC are seen in Figure 6.

##### Nanoparticles

The use of nanotechnology is rapidly gaining importance in medicine in general and is a wide subject of study for the treatment of HNSCC. Methods are being developed for the transmucosal delivery of drugs directly into the tumours using NPs. They are known to be biocompatible, biodegradable, and to allow for controlled drug release, which is essential for therapeutic efficiency [169]. Polymeric NPs have been widely investigated for chemoprevention, along with the drugs Cisplatin, Curcumin, Ellagic Acid, and 5-FU [170,171]. These agents have been encapsulated in chitosan nanoparticles and polymeric micelles to reduce systemic toxicities [171,172]. While there is potential for polymeric NPs as transmucosal DDS for HNSCC, they also come with the limitations of local cytotoxicity to other cells as well as difficulty in handling, which need to be further studied.

Inorganic NPs have also been tested and are sometimes preferred due to their better bioavailability and reduced toxicity [161]. Examples of inorganic NPs include gold, iron, and other metals like titanium. These NPs have a history of being used for diagnostic and imaging purposes, and their photo-thermal properties render them useful for therapeutic purposes [161,173,174]. Studies have shown that conjugated inorganic NPs require less energy to kill the malignant cells, which is a useful treatment goal [174]. Inorganic NPs have also been used in combination with photodynamic therapy to inhibit tumour proliferation and induce apoptosis [175]. They have also shown to be compatible with newer agents such as curcumin, as well as inhibitors targeting proteins that contribute to OSCC cell proliferation, to provide improved anti-cancer efficacy as well as prevent multidrug resistance [176,177].

To avoid issues of stability, biocompatibility, and long-term cytotoxicity, combinational NPs involving both polymeric and inorganic NPs are gaining importance as possible anti-cancer treatment options. These NPs have been investigated in HNSCC as delivery vehicles for conventional treatment options, especially with the advent of image-guided therapies [161,178]. Combinational NPs have noted applications in drug delivery based on external and internal stimuli that trigger drug release [179,180]. Common external stimuli include UV radiation, hyperthermia, x-radiation, ultrasound, and magnetic response [180]. Internal stimuli involve the spatio-temporal control of drug concentration levels based on pH responsiveness [180]. The use of combinational NPs in HNSCC, however, remains an area that is relatively unexplored, and the potential of these NPs needs to be further investigated as an option for transmucosal drug delivery. Table 4 shows, in detail, the studies that have employed various NPs for transmucosal administration in HNSCC.

##### Nanolipids

Conventional polymeric and inorganic NPs are known to be cytotoxic, especially when given through intratumoural injections, due to a limited uptake by the tumour cells and lack of space to load the agent [161,191]. Moreover, the reduced uptake also leads to the reduced efficiency of the drug itself. These aspects led to the shift of using lipid-based NPs, which are known for their high biodegradation levels [161]. Lipid-based NPs are usually in the form of dietary oils or fats and were initially used in the form of solid-based lipid NPs, which provided adequate controlled drug release for the local delivery of anticancer agents [191]. However, further research has shown that there was still only limited space to accommodate the drug into these NPs due to the solid crystalline structure [161]. Therefore, modified nanostructured lipid carriers (NLCs) were designed, which consist of both solid and liquid lipids that are embedded in a core matrix that allows for more of the agent to be loaded into the carrier [161]. A study by Fang et al. reported on the use of NLCs with curcumin for OSCC treatment [192]. They found that there was enhanced bioavailability of the drug, leading to better efficiency of curcumin when embedded in NLCs [192]. Other studies have shown the use of NLCs with conventional anticancer agents, like Docetaxel and Cisplatin, on HNSCC cell lines, as well as in mouse models harbouring these cancer cells, with reasonable success in demonstrating anti-tumour activity [193,194]. These NPs have shown promise in HNSCC settings and warrant further investigation.

##### Hydrogels

Hydrogels are three-dimensional structures that contain copious amounts of water and other biological fluids, which allow them to mimic the cellular or tumour microenvironment [161]. Due to this property, cells can be cultured in a three-dimensional environment as opposed to current conventional two-dimensional methods which do not provide a true representation of the microenvironment [195]. Hydrogels are also being used to encapsulate drugs and other biomolecules, even if they are hydrophilic [196]. They have many advantages over NPs since they can prolong the elimination of drugs from blood circulation and better therapeutic efficiency with improved intratumorally penetration [161]. Localized delivery using hydrogels for cancers works by altering their nanofibers to control how much of the drug is released [161,197]. Research has also shown that hydrogels play a significant role in tumour imaging, tissue regeneration and the creation of temporary prostheses [198].

Local intratumoural injections of chemotherapeutic agents have been tested; however, they were found to be quickly cleared by blood circulation [161]. The introduction of hydrogels has allowed for the controlled release of these agents, with less toxicity. An example of this is a study by Yang et al., where they used a self-healing chitosan hydrogel as a drug carrier for a hepatocarcinoma tumour in vivo [199]. They found that the hydrogel could adapt to the tumour microstructure and regenerate to prevent excess leakage of the drug into the blood circulation, as well as provide therapeutic effects [199]. Another notable property of hydrogels is their ability to co-load more than one therapeutic agent due to their protective nature. An example of this is shown by the study by Kim et al., where they proposed a hydrogel-based drug carrier for chemotherapeutic agents, Doxorubicin and 5-FU [200]. They showed that these co-loaded hydrogels could be injected smoothly into tumours in vivo [200]. Also, high concentrations of the drugs were present within the tumours and not distributed amongst the normal tissues, thus indicative of reduced systemic toxicities [200].

For HNSCC, the use of hydrogel-based drug carriers is being widely investigated, especially for oral tumours. Different types of hydrogels are being developed, due to the varying external environment of the oral cavity, such as changes in pH, photoelectricity, and temperature [201]. A pivotal study used an injectable, thermosensitive hydrogel encapsulating Cisplatin and Suberoylanilide Hydroxamic Acid (SAHA), a histone deacetylase inhibitor [202]. They demonstrated that the hydrogel was able to be sustained for two weeks in situ and could be injected smoothly into OSCC tumour-bearing mice with sustained release [202]. This paved the way for further studies to investigate thermosensitive hydrogels with different anticancer agents.

Photostimulation is a convenient method to be used with hydrogels because it controls drug release by controlling light and irradiation exposure [201]. Wu et al. developed an injectable, light-responsive hydrogel-system, made from mesoporous silica NPs with doxorubicin [203]. The hydrogel contained a green cyanine dye which could induce photothermal effects on the tumour cells [203]. The investigators demonstrated the synergistic effects of both chemotherapy and phototherapy in vitro and in vivo with less toxicity [203]. Enzyme-responsive hydrogels are also being investigated, especially using matrix metalloproteinases (MMP), as they are widely expressed in different oral tumours [201]. MMP-2-sensitive hydrogels have been developed by various investigators over the past couple of years, showcasing the faster degradation of the hydrogel in the tumour site [204]. To further strengthen the effectiveness of hydrogels, a combination of photo-sensitive and enzyme-responsive hydrogels has been tested successfully. Wang et al. designed a hydrogel system containing doxorubicin and green cyanine, which was MMP-2 sensitive [205]. They found that drug release was sustained in the presence of MMP-2 and was photosensitive, to inhibit the viability and metastasis of oral carcinoma cells in vitro [205]. These advances make hydrogels a promising drug carrier for transmucosal drug delivery; however, ways to prevent initial burst and improve mechanical properties will make it an even better treatment alternative [161].

##### Exosomes

Exosomes are extracellular vesicles that are secreted by many types of cells, including tumour and immune cells [161,206,207]. They are made up of constituents such as nucleic acids, protein conjugates, and lipids and are known to help in remodelling the extracellular matrix [161,206,208]. They have also demonstrated multiple roles in both promoting as well as suppressing cancers, which make them unique [207]. They can bypass immune surveillance and are natural carriers of cytotoxic drugs for cancer therapy [207]. They have been used as drug carriers with Paclitaxel, Curcumin, and Doxorubicin, but mainly through intravenous injections [161,209]. The main disadvantages of exosomes occur when given systemically, where they produce immune reactions and rapid accumulation into the liver and spleen [210]. They are also known to deliver only a limited dosage of therapeutic agents. Methods to encapsulate these exosomes are gaining traction, especially in cancer treatment [210].

Although research has shown that exosomes contribute to metastasis and drug resistance in HNSCC, there are situations where they can also be useful as drug delivery vehicles [206]. Their bi-layer structure and biocompatibility allows them to interact with the tumour cells [206]. A study by Cohen et al. compared the use of MSC-derived exosomes with tumour-bearing exosomes as potential drug vectors and they found that the former had better penetration and distribution in the tumours, which shows that the exosome type plays a better role prior to testing in HNSCC [211]. Clinical trials are also underway in pancreatic cancers using MSC-exosomes containing SiRNA to target oncogenic mutations [212]. Trials using exosomes are yet to materialize in head and neck settings; however, in vitro studies using MSC-derived exosomes to restore salivary gland function after radiation have been conducted with reasonable success [213]. In general, since exosomes also play a role in tumour proliferation, further research is required to investigate how best to use them as transmucosal drug delivery vehicles.

## 4. Conclusions

HNSCC requires a multidisciplinary approach, which is effective but can simultaneously affect the patients’ quality of life. Chemoradiation is associated with toxic side effects and warrants alternative treatment strategies. This review focused on the various LDDS that have been tested for the treatment of HNSCC, as well as for the delivery of agents to mitigate the side effects of chemoradiation and immunotherapy. It is imperative to find treatment methods that avoid systemic circulation and LDDS can do this through the rapid entry of agents directly into the diseased area. This review demonstrated the potential of targeted drug delivery in HNSCC using technologies such as transdermal and transmucosal drug delivery.

TDD, although a systemic route, is a more localized route that delivers drugs through the skin, using either gels or transdermal patches. Over time, TDD has evolved with the introduction of PEs and micro-nanocarriers, which has allowed better penetration through the skin and further research with anticancer therapies. Microneedles are currently the TDD systems that have the most potential, as they can penetrate the stratum corneum painlessly and allow for rapid absorption and controlled release. Transmucosal drug delivery systems make use of the mucous membranes to deliver drugs, allowing them to be delivered at various sites. The various modes of administration, such as oral, intranasal, and rectal delivery, allow transmucosal drug delivery to be considered for a variety of applications. This DDS has proven applications in radiation-induced xerostomia, oral mucositis, as well as in novel applications in delivering anti-tumour drugs for HNSCC using different nanocarriers.

Both TDD and transmucosal systems have their own advantages over the other, such as the easily penetrable mucous tissue and better adhesiveness of the skin. However, the fact that both systems can evade systemic circulation makes them attractive alternate treatment strategies that have the potential to be translated into clinical practice. Most studies incorporating LDDS in HNSCC settings are still in the pre-clinical stage, therefore, it is important to continue exploring these treatment methods to reduce the side effects of chemoradiation and immunotherapy, as well as to treat HNSCC tumours.

## Figures and Tables

**Table 1:** Micro-nanocarriers used in transdermal drug delivery.

Micro-Nanocarriers	Agents Used	Applications	References
Liposomes	Doxorubicin	Ovarian Cancer Kaposi’s Sarcoma Multiple Myeloma HNSCC	[90,91,92]
Modified Liposomes (Ethosomes/Transferosomes)	5-FU Curcumin Doxorubicin	Melanoma HNSCC Non-small Cell Lung Cancer	[93,94,95]
Polymeric Nanoparticles	Abraxane Paclitaxel Doxorubicin 5-FU Cisplatin	Prostate Cancer Breast Cancer Skin Cancer HNSCC/OSCC (in vitro testing)	[96,97,98,99]
Inorganic Nanoparticles	Doxorubicin used in conjunction with physical enhancers (e.g., hyperthermia, thermal ablation	Liver Cancer Breast Cancer Prostate Cancer Radiotherapy/Diagnostic Imaging	[100,101,102]

HNSCC—head and neck squamous cell carcinoma; OSCC—oral squamous cell carcinoma; 5-FU—5-Fluorouracil.

**Table 2:** Transmucosal routes and applications.

Routes	Mode	Agents Used	Applications	References
Intranasal	Nasal Spray Atomization Devices Nasal Powders Chitosan NPs	Fentanyl Sodium Nicotine Replacement Calcitonin Oxytocin Desmopressin	Acute Pain Management for Cancers Nasal Congestion/Rhinitis Nicotine Replacement Breast-feeding Bleeding Control	[125,126,127,128,129,130,131]
Rectal	Chitosan-based carriers	5-FU	Colon Cancers	[132,133]
	Gelatin hydrogels	Docetaxel		
Oral	Buccal/Sublingual patches	Fentanyl Sodium	HNSCC (OSCC)—see Table 3 for detailed uses, references.	
	Tablets/Lozenges	Pilocarpine, Xylitol		
	Mouthwashes/Sprays	Triamcinolone Acetonide		
	Hydrogels	Clonidine Lauriad Chamomile Extract Cisplatin Curcumin 5-FU Doxorubicin Paclitaxel		

5-FU—5-Fluorouracil; HNSCC—head and neck squamous cell carcinoma; OSCC—oral squamous cell carcinoma.

**Table 3:** Applications of oral transmucosal drug delivery systems in HNSCC.

Applications	Method	Agents Used	References
Pain Management	Lozenges mucoadhesive tablets	Fentanyl Citrate	[79]
Radiation-induced xerostomia	Mouthwash	Pilocarpine Dietary supplements Xylitol/Adipic Acid	[147,148,149,150,151,152,153]
	Lozenges Mucoadhesive polymers (buccal inserts) Nanofiber mats (Intradermal)		
Oral Mucositis	Mucoadhesive hydrogels Mucoadhesive films Mucoadhesive buccal tablets Mucoadhesive topical gel	MuGard Triamcinolone Acetonide Clonidine Lauriad Chamomile	[25,154,155,156,157,158,159,160]
Anti-tumour Response	Nanocarriers (Nanoparticles, Nanolipids, Hydrogels, Exosomes)	Cisplatin, Curcumin, 5-FU, Ellagic Acid, Doxorubicin, Quinacrine, Docetaxel, Paclitaxel	[161,162,163]

5-FU—5-Fluorouracil.

**Table 4:** Prominent studies using transmucosal nanoparticles in HNSCC.

Nanoparticle Type	Agent/Metal Used	Study	Result	References
Polymeric Nanoparticles	Cisplatin	Evaluate safety and efficacy of polymeric micelles (in vitro, in vivo)	Reduced nephrotoxicity in mice, demonstrated anti-tumour activity on OSCC cell lines.	Endo et al. [171]
	Cisplatin	In vivo studies and clinical trials use chitosan particles to evaluate safety and efficacy.	In vivo retention of Cisplatin in tumours; 69% tumour reduction in clinical trials with no side effects.	Goldberg et al. [172]
	Curcumin	Use of chitosan-coated nanoparticles in vitro on SCC-9 oral cancer cell lines.	Curcumin-loaded nanoparticles showed reduced cell viability and cytotoxicity.	Mazzarino et al. [181,182]
	Ellagic Acid	Chitosan Nanoparticles encapsulated with Ellagic Acid	Sustained release and increased cytotoxocity of Ellacgic Acid in vitro on OSCC cell lines.	Arulmozhi et al. [183]
	5-FU	PLGA Nanoparticles with 5-FU in vitro/in vivo	Prolonged release of 5-FU with increased oral bioavailability; reduced GI side effects in vivo.	Li et al. [184]
Inorganic Nanoparticles	Gold	EGFR-conjugated nanoparticles with photothermal energy on OSCC cell lines.	Less production of energy required, suggestive that less photothermal energy required to kill malignant cells.	El Sayed et al. [174]
	Titanium	Photodynamic therapy combined with Titanium Nanoparticles in vitro/in vivo.	Allowed for deeper penetration in malignant tissues, inhibition of tumour proliferation, apoptosis when targeting EGFR.	Lucky et al. [175]
	Iron	In vitro/in vivo studies using supramagnetic nanoparticles for combined chemotherapy/hyperthermia.	Induction of cancer cell death and better stability by using AMF; good, controlled drug delivery, therapeutic efficacy, and reduced toxicity.	Sato et al. [185]
	Iron	CD44 supramagnetic nanoparticles on Cal-27 oral carcinoma cell lines, in vivo tumours.	Good biocompatibility, AMF induced cell death; magnetic hyperthermia inhibited the growth of tumours in mice.	Su et al. [186]
	Silica-Bleomycin	Good biocompatibility, AMF induced cell death; magnetic hyperthermia inhibited the growth of tumours in mice.	Agents were retained to the focal area when a magnetic field was applied, sustainable release; tumour growth suppressed in vivo.	Zhang et al. [187]
	Silica-Doxorubicin	Nanoparticle containing Doxorubicin and SiRNA for HNSCC	Induction of apoptosis and reduced expression of MDR1, reduction in tumour size in vivo by 81.64%	Wang et al. [188]
	Silica	Mesoporous Nanoparticles with MTH1 inhibitor and SiRNA on Cal-27 oral cancer cells	Controlled drug release, cytotoxicity and apoptosis increased, reduction in tumour burden in vivo.	Shi et al. [176]
Combinational Nanoparticles (Polymeric inorganic)	PLGA-Silver Nanoparticles	Hybrid Nanoparticles containing Quinacrine on OSCC cell lines and OSCC stem cells.	Nanoparticles able to exhibit cytotoxic behaviour and prevent angiogenesis.	Satapathy et al. [189]
	Quinacrine-based Silver and Gold Hybrid Nanoparticles	Mechanism of anti-cancer activity in vitro/in vivo on OSCC using hybrid nanoparticles.	Low toxicity levels in SCC-9 Oral Cancer cell lines, inhibited DNA replication by reducing topoisomerase activity; reduction in tumour volume and bodyweight recovery in vivo.	Hembram et al. [190]

5-FU—5-Flurouracil; OSCC—oral squamous cell carcinoma; HNSCC—head and neck squamous cell carcinoma; PLGA—poly lactic-co-glycolic acid; EGFR—epidermal growth factor receptor; CD44—cluster of differentiation 44; AMF—altering magnetic field; MDR1—multidrug resistance protein 1; MTH1—MuT homolog 1.

## Data Availability

Not applicable.
